# From isolation to application: a case study of arbuscular mycorrhizal fungi of the Arabian Peninsula

**DOI:** 10.1007/s13199-021-00824-x

**Published:** 2021-12-08

**Authors:** Mohamed N. Al-Yahya’ei, Janusz Błaszkowski, Hamood Al-Hashmi, Khaled Al-Farsi, Ismail Al-Rashdi, Annette Patzelt, Thomas Boller, Andres Wiemken, Sarah Symanczik

**Affiliations:** 1 Zurich Basel Plant Science Center, Institute of Botany, University of Basel, Hebelstrasse 1, CH-4056 Basel, Switzerland; 2 Directorate General of Agricultural and Livestock Research, Ministry of Agriculture, Fisheries and Water Resources, P. O. Box 50, P.C. 121, Muscat, Sultanate of Oman; 3 Department of Integrative Agriculture, College of Agriculture and Veterinary Medicine, United Arab Emirates University, 15551, Al Ain, United Arab Emirates; 4 Oman Animal and Plant Genetic Resources Center (Mawarid), Ministry of Higher Education, Research and Innovation, P.O. Box 515, P.C. 123, Muscat, Sultanate of Oman; 5 Department of Plant Protection, West Pomeranian University of Technology, Szczecin, Slowackiego 17, 71434 Szczecin, Poland; 6 Oman Botanic Garden, P. O. Box 808, P.C. 122, Muscat, Sultanate of Oman; 7 Research Institute of Organic Agriculture (FiBL), Ackerstrasse 113, 5070 Frick, Switzerland

**Keywords:** Date palm, Desert ecosystem, Mycorrhizal symbiosis, Oman, Native plants

## Abstract

The vegetation in the Arabian Peninsula experiences drought, heat, soil salinity, and low fertility, mainly due to low phosphorus (P) availability. The beneficial mycorrhizal symbiosis between plants and arbuscular mycorrhizal fungi (AMF) is a key factor supporting plant growth under such environmental conditions. Therefore, AMF strains isolated from these soils might be useful as biotechnological tools for agriculture and revegetation practices in the region. Here we present a pioneering program to isolate, identify, and apply AMF isolated from rhizosphere soils of agricultural and natural habitats, namely date palm plantations and five native desert plants, respectively in the Southern Arabian Peninsula. We established taxonomically unique AMF species as single-spore cultures as part of an expanding collection of AMF strains adapted to arid ecosystems. Preliminary experiments were conducted to evaluate the abilities of these AMF strains to promote seedling growth of a main crop *Phoenix dactylifera* L. and a common plant *Prosopis cineraria* L. (Druce) in the Arabian Peninsula. The results showed that inoculation with certain AMF species enhanced the growth of both plants, highlighting the potential of these fungi as part of sustainable land use practices in this region.

## Introduction

The application of arbuscular mycorrhizal fungal (AMF) inocula in agriculture and revegetation programs has recently become more prominent, largely due to the increasing number of studies demonstrating improved plant growth after inoculation (Igiehon and Babalola 2017; Bano and Uzair 2021). The application of AMF inocula at the nursery stage in horticulture is especially beneficial as it provides the plants with an established, tailored AM symbiosis before they are transplanted in the field (Nzanza et al. 2012; Ren et al. 2019). Hence, colonization of the soil by fungal hyphae can start immediately (Jeffries et al. 2003). AMF hyphae have been shown to be important infection structures in arid and semi-arid ecosystems (Requena et al. 1996; Azcón-Aguilar et al. 2003). Therefore, the application of AMF in the nursery increases the infectivity of soils in such habitats (Requena et al. 2001).

AMF species or strains adapted to distinct environmental conditions have been described previously (Marulanda et al. 2007; Lekberg and Koide 2008; Antunes et al. 2011). Antunes et al. (2011) observed that AMF exhibit optimal performance in experimental conditions that closely resemble the environmental conditions from where they were derived. Moreover, Bauer et al. (2020) have shown that plant community productivity responded positively to local adaptation of AMF to soil conditions yielding in higher aboveground biomass. Further, it has been shown that inoculation with exotic AMF may disturb the structure of native AMF communities (Mummey et al. 2009; Koch et al. 2011; Symanczik et al. 2015). Therefore, possible changes in native AMF communities via invasion of exotic ones should be considered to assess the risk of future unintended consequences (Schwartz et al. 2006).

Here, we describe the establishment of a culture collection of single-spore derived AMF strains, originally recovered from different habitats and a variety of native plant species in Oman (Al-Yahya’ei et al. 2011; Symanczik et al. 2014a, b). The AMF culture collection provides a crucial basis for mycorrhizal research under the harsh conditions of the Southern Arabian Peninsula (Symanczik et al. 2015), which is also an environmentally unique landscape (Fisher and Membery 1998; Glennie and Singhvi 2002).

Two plant species, *Phoenix dactylifera* L. (date palm) and *Prosopis cineraria* L. (Druce) (local name: Ghaf) were selected for inoculation with AMF species from the culture collection. Date palm is the main crop in Arabia and in many other desert areas of the world (Zaid and De Wet 2002), and is considered a survival crop due to the high nutritional value of its fruit (Al-Shahib and Marshall 2003). *P. cineraria* is a tree native to the Arabian Peninsula, Iran, Afghanistan, Pakistan, and India, and plays an important role as an agro-forestry species as it forms a tripartite symbiosis with AMF and rhizobial nitrogen-fixing bacteria.

The aims of this study were to isolate, identify, propagate, and functionally evaluate the AMF from agricultural and natural habitats in the Southern Arabian Peninsula. Such an approach can help integrate efficient and native AMF strains into sustainable agriculture and revegetation programs on the Arabian Peninsula.

## Materials and methods

### Establishment of the AMF culture collection

The isolated species of AMF recovered from the Al-Sharqiya region of Oman are listed in Table 1. Four sites were sampled. Two sites were date palm plantations that were established on a sandy plain. Management followed local traditional farming practices at one site and modern agriculture practices at the other. A ruderal plant, *Polygala erioptera* DC, growing on the dry strip between the palm trees at the modern agriculture site was also included in the sampling. A third sampled site was an undisturbed habitat adjacent to the modern date palm plantation. The natural vegetation of this habitat consisted mainly of three perennial plant species, *Tetraena qatarensis*, *Salvadora persica* L., and *Prosopis cineraria*, and open vegetation with dispersed patches of dry grass apparently growing after rainfall. The fourth sampling site was in the Al-Sharqiya Sands, a large sand desert in northern Oman (Patzelt 2015). A native plant, *Heliotropium bacciferum* Forssk., growing in this habitat was included in the samples. The geography of the region, sampled sites, environmental conditions, and soil properties are described in Al-Yahya’ei et al. (2011) and Symanczik et al. (2014a, b). Trap cultures were established in each of the four sampling sites to collect soil and root samples of plants. The trap cultures and greenhouse conditions for subsequent cultures have been described previously (Al-Yahya’ei et al. 2011).

**Table 1:** Number of arbuscular mycorrhizal fungal (AMF) single-spore-derived cultures in relation to their original host plants and the host plants’ habitat

AMF species	Associated plant species	Habitat	Number of AMF cultures
*Claroideoglomus drummondii*	*Tetraena qatarense* *Salvadora persica* *Prosopis cineraria* Inter-plant area	Undisturbed habitat	1 6 8 8
*Desertispora omaniana*	*Tetraena qatarensis* *Salvadora persica* Inter-plant area	Undisturbed habitat	1 1 2
*Diversispora aurantia*	*Prosopis cineraria* *Phoenix dactylifera*	Undisturbed habitat Traditional date palm plantation	3 3
*Diversispora spurca*	*Tetraena qatarensis* *Salvadora persica* *Prosopis cineraria* Inter-plant area *Heliotropium bacciferum* *Polygala erioptera* *Phoenix dactylifera* *Phoenix dactylifera*	Undisturbed habitat Undisturbed habitat Undisturbed habitat Undisturbed habitat Sand dunes Undisturbed habitat Modern date palm plantation Traditional date palm plantation	9 16 4 3 3 4 1 3
*Pervetustus simplex*	*Tetraena qatarense* *Salvadora persica*	Undisturbed habitat	3 2
*Rhizophagus arabicus*	Inter-plant area	Undisturbed habitat	2
*Septoglomus africanum*	*Phoenix dactylifera*	Traditional date palm plantation	4
*Septoglomus nakheelum*	*Phoenix dactylifera*	Traditional date palm plantation	3
**Total number of AMF cultures**			**90**

### Inocula preparation and identification

Freshly produced spores from the trap cultures were used to established more than 1000 single-spore assays to derive single-spore cultures, as described previously (Symanczik et al. 2014a, b). Single-spore assays were checked for sporulation, and positive cultures were used as inocula for further propagation by culturing with a consortium of *Allium porrum* L., *Hieracium pilosella* L., and *Plantago lanceolata* L. as AMF host plants. The resulting mycorrhizal inocula were identified using morphological and molecular identification methods, as described previously (Symanczik et al. 2014a, b; Blaszkovski et al. 2017; Symanczik et al. 2018).

### Inoculation of date palm

The date palm inoculation experiment was conducted in the Agricultural Research Station of Jemah, Oman Ministry of Agriculture, Fisheries and Water Resources. The date palm seedlings (Khalas Al Daherah variety) were tissue-cultured. Seedlings were established for 28 months at the callus stage under tissue culture conditions, then transplanted into 100-mL pots filled with peat moss (Plantafior, Germany) and vermiculite (1:1; v/v) and grown for 5 months in a shade house (temperature: 24–32 °C; relative humidity: 65–70%). Date palm seedlings for the experiment were selected based on their homogeneity of total height and leaf number. Six AMF strains were used as single-species inocula, *Diversispora aurantia* (strain G8), *Septoglomus africanum* (strain G14), *Claroideoglomus drummondii* (strain F41), *Desertispora omaniana* (strain F69), *Pervetustus simplex* (strain C49), and *P. simplex* (strain C57). One consortium inoculum that included all five species was also used. The inocula, added as substrate inocula, included a total of 250 spores and a volume of 15 g pre-weighed into 15 ml reaction tubes. Those single-strain inocula with high spore numbers were diluted with sterilized inocula to reach a total volume of 15 g. While transplanting the seedlings into 3-L plastic bags filled with peat moss (Plantafior, Germany) and vermiculite (1:1; v/v) the inocula was homogeneously spread onto the root system. Two controls were included: a non-mycorrhizal (NM) control, to which no AMF inoculum was added, and a non-mycorrhizal fertilized (NM-fert) control that was the only treatments receiving foliar-fertilizer (Micromix X200 SP, Pioneers Chemicals Factory CO, SA) weekly. The latter represented the conventional handling practice of date palm seedlings at the Research Station. Plant growth performance after 12 months was estimated by measuring the total lengths of all leaves of a single plant (accumulated length of all leaves as an estimate of biomass) and the number of leaves. No destructive harvest was performed as plants were later transplanted to the field.

### Inoculation of P. cineraria

The inoculation experiment was conducted at the Oman Botanic Garden (www.omanbotanicgarden.com; Patzelt et al. 2008). *P. cineraria* seeds were soaked in water overnight before being sown in compartmented germination trays. Each seed was placed in a 50-mL pot filled with a mixture of peat moss (Plantafior, Germany) and vermiculite (1:1; v/v) and supplemented either with mycorrhizal inocula or one of the control treatments. Seven single-species inocula were used, *P. simplex* (strain C49), *Septoglomus nakheelum* (strain G90), *Di. spurca* (strain K46), *Rhizophagus arabicus* (strain F80), *De. omaniana* (strain F69), *Di. aurantia* (strain G5), and *S. africanum* (strain G14). In addition, four different consortia were used as inocula. These were consortium 1 (*P. simplex* strain C49 and C56), consortium 2 (*C. drummondii* strain F41 and *De. omaniana* strain F69), consortium 3 (*S. africanum* strain G14 and *De. omaniana* strain F69), and consortium 4 (*P. simplex* strain C49, *C. drummondii* strain F41, *S. africanum* strain G41, and *De. omaniana* strain F69). Additionally, four non-mycorrhizal control treatments were applied: microbial wash (control 1), autoclaved inoculum carrier (control 2), microbial wash and autoclaved inoculum carrier (control 3), and a negative control (control 4). Eight seeds were used as replicates for mycorrhizal treatments and 20 seeds for the control treatments. Higher numbers of seeds in the control treatments were used as there were low germination rates in preliminary experiments (data not shown). For inoculation, 5 g of substrate inocula containing a total of 250 spores was homogeneously mixed into the growing substrate using a sterile spatula. Those single-strain inocula with high spore numbers were diluted with sterilized inocula to reach a total volume of 5 g. Each pot, except control 2 and control 4, received 5 mL of the microbial wash to correct possible differences in microbial communities (Koide and Elliott 1989). The microbial wash was prepared by wet-sieving 100 g of each inoculum through a 32-µm sieve and a paper filter (FS 14 1/2; Schleicher & Schuell, Whatman plc, United Kingdom), yielding a final volume of 1 L. After 1 month, seedlings were transplanted into 300-mL pots filled with a mixture of original soil from the surrounding area of the Botanic Garden and fine peat moss (1:1; v/v), and grown for 2 months under greenhouse conditions (temperature: 25–28 °C; relative humidity: 60–70%). Pots were placed on a greenhouse bench and carefully irrigated by hand to avoid contamination between treatments.

Germination rates were calculated as the percentage of surviving seedlings divided by the initial number of seeds sown (Table 2). Plant growth performance was estimated 3 months after sowing by measuring the total length of all branches of a single plant (accumulated length of all branches as an estimate of total biomass). Survival rates were calculated as the percentage of seedlings present after 12 months divided by the number of germinated seedlings. No destructive harvest was performed as plants were later transplanted to the field.

**Table 2:** Germination of seeds and survival of seedlings of *Prosopis cineraria* under different control and inoculation treatments

Arbuscular mycorrhizal fungal treatment	Initial seeds treatment^−1^	Germination of seeds		Survival of seedlings	
		Number	%	Number	%
Control 1	40	7	18	7	100
Control 2	40	12	30	8	67
Control 3	40	13	33	7	54
Control 4	40	10	25	6	60
*Pervetustus simplex* strain C49	8	5	63	5	100
*Septoglomus nakheelum*	8	5	63	5	100
*Diversispora spurca*	8	5	63	5	100
*Rhizophagus arabicus*	8	5	63	5	100
*Desertispora omaniana*	8	5	63	5	100
*Diversispora aurantia*	8	4	50	4	100
*Septoglomus africanum*	8	7	88	7	100
Consortium 1	8	6	75	6	100
Consortium 2	8	3	38	3	100
Consortium 3	8	5	63	5	100
Consortium 4	8	4	50	4	100

Consortium 1 (*P. simplex* strain C49 and C56), consortium 2 (*C. drummondii* and *De. omaniana*), consortium 3 (*S. africanum* and *De. omaniana*), consortium 4 (*P. simplex* strain C49, *C. drummondii, S. africanum, and De. omaniana*), control 1 (microbial wash), control 2 (autoclaved inoculum carrier), control 3 (microbial wash and autoclaved inoculum carrier), control 4 (no amendments)

### Statistical analyses

Data were analyzed using one-way ANOVA followed by Tukey’s honest significant difference test with a significance level of a D 0.05. Normality of residuals was tested using Shapiro–Wilk test. Analyses were performed using JMP software version 11 (SAS, Cary, NC, United States).

## Results & Discussion

### Establishment of the AMF culture collection

**Fig. 1:**
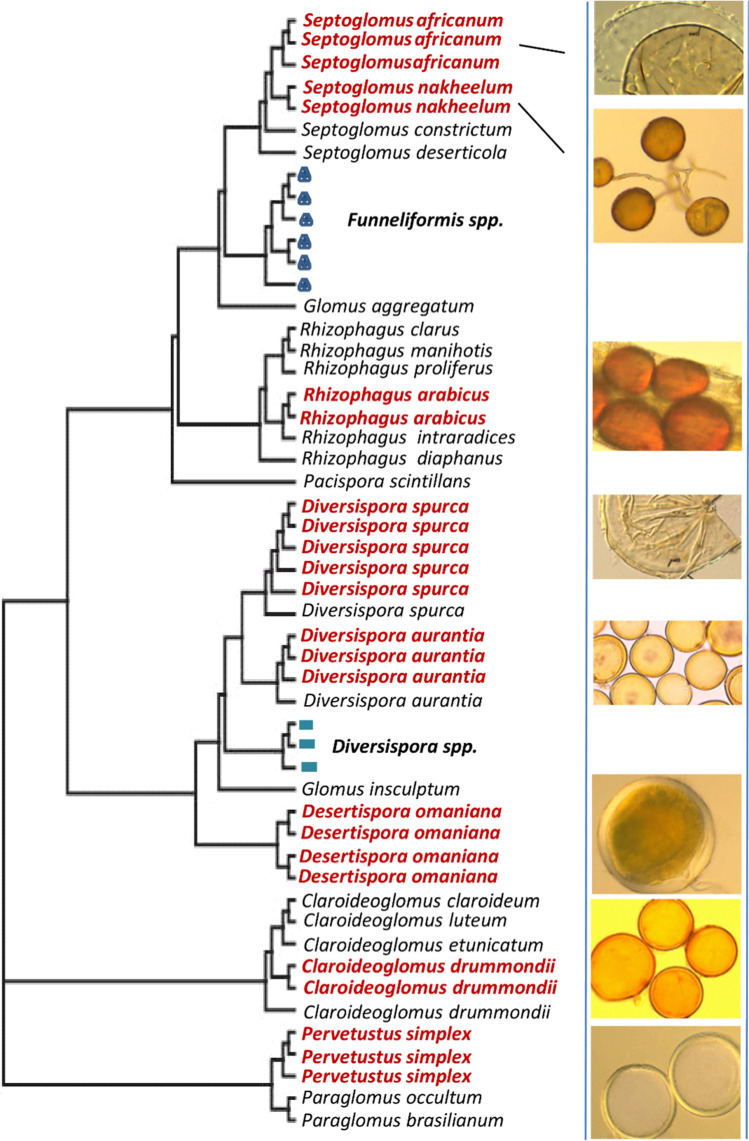
Phylogenetic positions and morphology of the eight arbuscular mycorrhizal fungal (AMF) species recovered from the Arabian Peninsula. The simplified phylogenetic tree was inferred from the partial LSU rDNA region (628 dataset characters), and shows the positions of the isolated AMF species (shown in colored boldface). An image of each of the AMF species is associated with its phylogenetic position. Sequences were aligned in PAUP*4b10 (Swofford 2001) to other sequences from GenBank of species within the same genera. The phylogenetic tree was inferred using maximum likelihood criteria as implemented in PAUP*

Of more than 1000 single-spore assays, only 90 single-spore derived cultures were successfully established. Amongst them, eight different AMF species were identified. Four were already described AMF species, including *Claroideoglomus drummondii, Diversispora aurantia, Diversispora spurca,* and *Septoglomus africanum* (Symanczik et al. 2014b). However, four species were previously unknown. They were given names unique to the geographical region of the Southern Arabian Peninsula: *Desertispora omaniana, Rhizophagus arabicus, Septoglomus nakheelum* and *Pervetustus simplex* (Symanczik et al. 2014a; Blaszkovski et al. 2017). The phylogenetic and morphological characterization of all eight species is represented in Fig. 1. Numbers of established AMF single-spore cultures in relation to their original host plant species are shown in Table 1.

The importance and significance of establishing a culture collection specific to a target ecosystem has been previously demonstrated for degraded semi-arid Mediterranean ecosystems (Barea et al. 2011). Similar efforts were made at the Centre for Mycorrhizal Culture Collection (CMCC) which houses and maintains cultures from different agro-ecological zones in India (TERI) and for AMF in China (Gai et al. 2006). Culture collections of AMF from specific ecosystems are fundamental to the process of applying these beneficial fungi in revegetation programs. Koziol et al. (2018) recently reviewed the potential and benefits of applying native AMF for restoration efforts with a focus on grasslands. The importance of native AMF as keystone taxa for revegetation was recently highlighted also by Qin et al. (2019). Their field study on the Tibetan plateau revealed the significance of AMF in enhancing bacterial and fungal richness and diversity, soil structure stability, and nutrients cycling.

In the Southern Arabian Peninsula, the Oman Botanic Garden holds the largest documented collection of Arabian plants in the world (Patzelt et al. 2008, 2009). The garden aims to propagate and display the complete indigenous flora of the Sultanate of Oman, and address the urgent need for conservation solutions to the biodiversity crisis. Mycorrhizal biotechnology might be a crucial factor to overcome the difficulties in propagation and maintenance of some indigenous plants.

### Growth performance of inoculated date palms

**Fig. 2:**
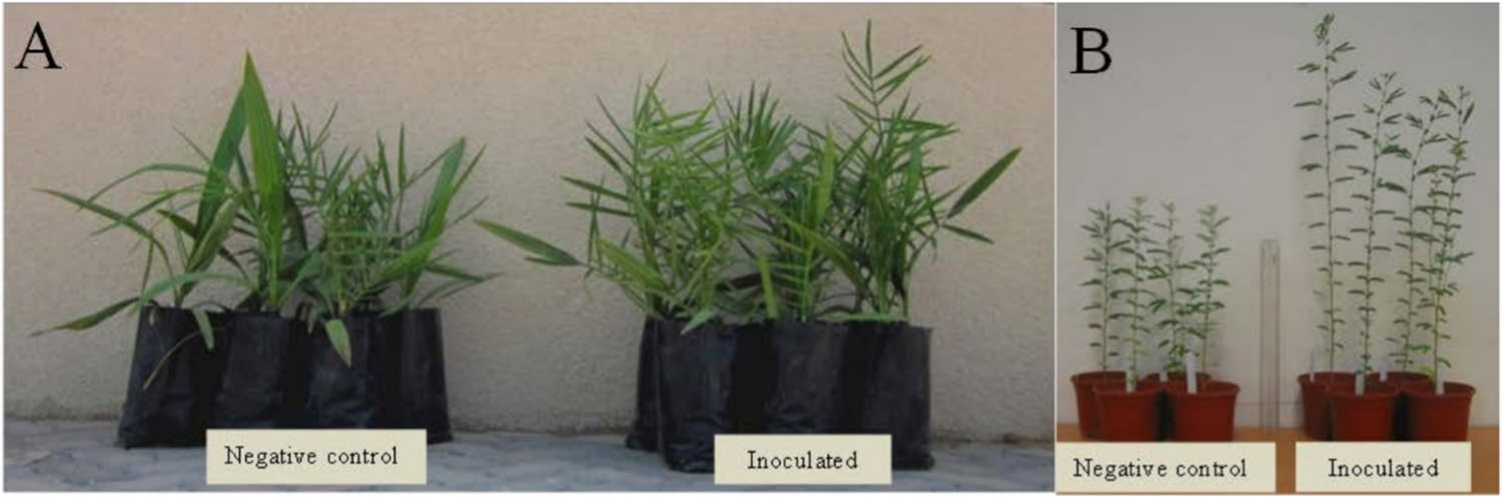
Effect of inoculation with arbuscular mycorrhizal fungi (AMF) on plant growth; (a) date palms grown as non-mycorrhizal control (left) and as mycorrhizal plants, inoculated with *Diversispora aurantia* (right); (b) *Prosopis cinerari*a grown as non-mycorrhizal control (left) and as mycorrhizal plants, inoculated with *Septoglomus africanum* (right)

**Fig. 3:**
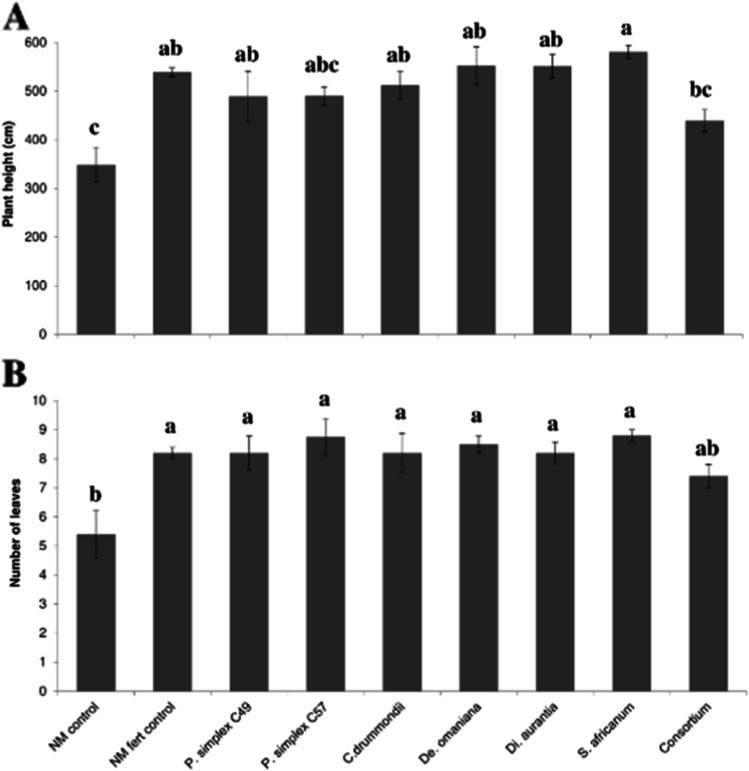
Impact of inoculation with different arbuscular mycorrhizal fungal (AMF) species on (a) total leaf length and (b) the number of leaves of date palm seedlings. The consortium included all five AMF species: *Diversispora aurantia, Septoglomus africanum, Claroideoglomus drummondii, Desertispora omaniana,* and *Pervetustus simplex*. Different letters above bars indicate significant differences according to Tukey’s honest significant difference test (P ≤ 0.05). Data represent means ± standard error (n = 4–5)

The growth of date palm seedlings (total leaf length: F (8,34) = 5.9, P > 0.0001; numbers of leaves: F (8,34) = 4.3, P = 0.0012) was significantly affected by AMF inoculation. The total leaf length of date palms were significantly higher for all AMF strains and the NM-fert control plants except *P. simplex* strain C57 and the consortium showing similar values as the NM-control plants (Fig. 2A and Fig. 3A). Also the number of leaves was significantly higher in all AMF treatments and in the NM-fert control plants except the AMF consortium compared with that in NM-control plants (Fig. 3B). Similar growth of mycorrhizal date palm seedlings compared to NM-fert control seedlings occurred without the application of mineral fertilizers, which may be attributed to the integration of AMF inocula into the propagation process. Inoculation with *S. africanum* even resulted in better growth, as reflected in total leaf length, compared to the NM-fert control. Thus, by introducing AMF early in the propagation process, plants grew better without the need for additional fertilizers. The two AMF species that showed the greatest effect on total leaf length, *Di. aurantia* and *S. africanum*, were originally recovered from date palm plantations (Symanczik et al. 2014b) and might have been specially adapted to interact with date palms.

The results are in accordance with previous studies investigating the interaction of AMF and date palms (Anli et al. 2020a, b; El Kinany et al. 2019). Tissue-cultured date palm seedlings inoculated with a commercial AMF inoculum showed enhanced growth, and mycorrhizal seedlings grew better without fertilizer than non-mycorrhizal fully-fertilized control seedlings (Shabbir et al. 2011), which is in accordance with these results. Similarly, Baslam et al. (2014) observed that inoculation of date palm seedlings with *Rhizophagus intraradices* increased leaf area, root length and shoot and root dry weight. Meddich et al. (2018) have also shown that shoot area and shoot dry weight was increased when seedlings were inoculated with different AMF species or with a native AMF complex isolated from a date palm grove in Morocco.

Date palms are commonly propagated by tissue culture techniques. After in-vitro plant establishment, the seedlings are transferred to artificial growth substrates (peat, vermiculite) that lack AMF. At this stage, mineral fertilizers are used to increase seedling growth. After being transplanted into the field, it is difficult for date palms to establish AMF symbiosis due to the low infect ion potential of most desert soils (Requena et al. 2001) and sometimes due to the high amounts of fertilizer added to the nutrient deficient soils (Smith and Read 2008). If date palm seedlings could established AMF symbiosis prior to being transplanted into the field, the high input rates of mineral fertilizers could be reduced, while achieving the same growth and yield, as shown for oil palms (Schultz 2001). Another problem related to field transplantation is the low survival rate of date palm seedlings (Zaid and De Wet 2002). Integration of AMF into the propagation process might improve the low success rates, as shown in oil palms, in which survival rate increased from 55% for non-mycorrhizal oil palm seedlings to 83% and 100% for mycorrhizal oil palm seedlings (Schultz 2001).

### Growth performance of inoculated P. cineraria

**Fig. 4:**
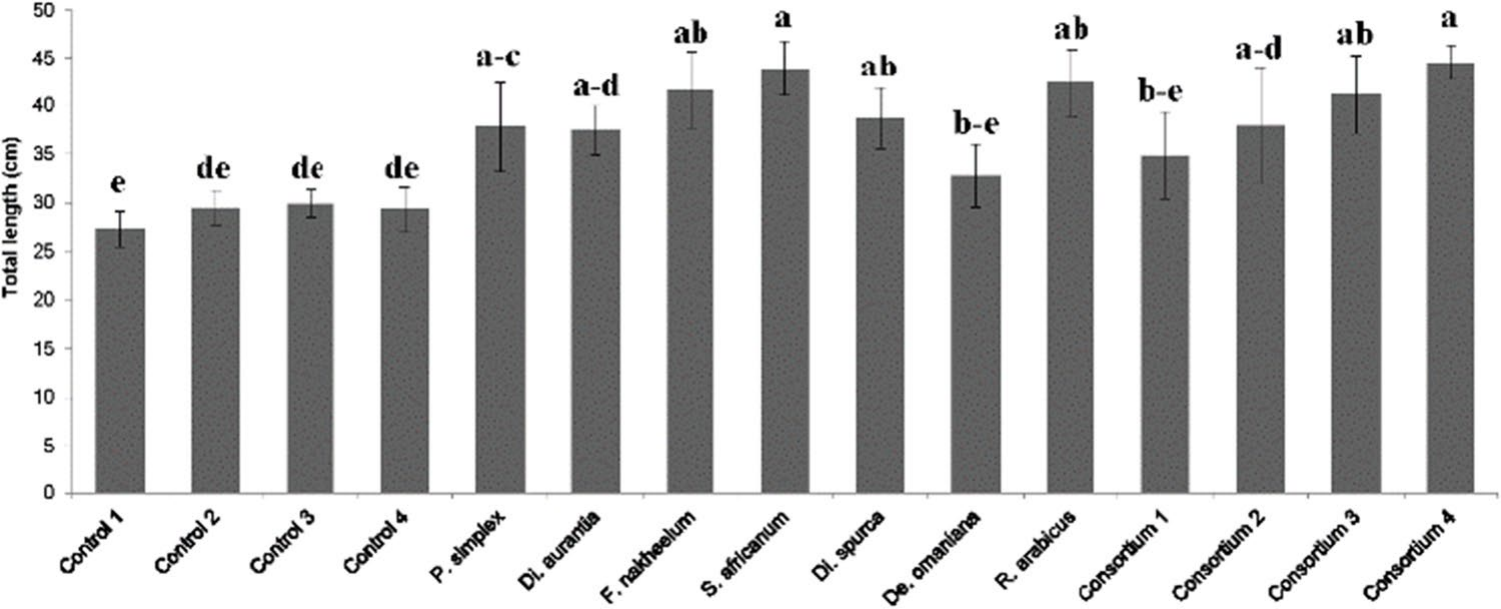
Impact of inoculation with different arbuscular mycorrhizal (AM) fungal species on the height of *Prosopis cineraria* seedlings 3 months after sowing compared with the growth of non-mycorrhizal controls: microbial wash (control 1), autoclaved inoculum carrier (control 2), microbial wash and autoclaved inoculum carrier (control 3), and negative control (control 4). In addition to inoculation with single AMF species, four consortia were used as inocula: consortium 1 (*Pervetustus simplex* strain C49 and C56), consortium 2 (*Claroideoglomus drummondii* and *Desertispora omaniana*), consortium 3 (*Septoglomus africanum* and *De. omaniana*), and consortium 4 (*P. simplex* strain C49, *C. drummondii; S. africanum,* and *De. omaniana*). Different letters above bars indicate significant differences according to Tukey’s honest significant difference test (P ≤ 0.05). Data represent means + SE (n = 3–13)

Application of AMF inocula improved the germination and survival of *P. cineraria* during seedling establishment (Table 2). Germination and survival was considerably reduced, to a varying extent, for NM-control treatments, while many more seeds germinated in the mycorrhizal treatments, except for consortium 2 (*C. drummondii* and *De. omaniana*), which had a germination rate of 38%. In addition, all mycorrhizal seedlings survived in contrast to almost all control treatments (Table 2). The growth of *P. cineraria* was significantly affected by AMF inoculation (F (14,87) = 4.165, P < 0.001). After 3 months of growth, seedling height was significantly enhanced in 9 of the 11 mycorrhizal treatments compared with that in all control treatments (Fig. 2b and Fig. 4). Among these, inoculation with *S. africanum* and the consortium 4, which included most of the single strains, achieved the highest shoot length.

The application of AMF under nursery conditions has been successfully demonstrated for a range of plants including vegetables, spices, fruit crops, tropical plantation crops as well as ornamental crops and trees (Azcón-Aguilar and Barea 1997, Koltai et al. 2008, Chapdelaine et al. 2008, Baum et al. 2015, Symanczik et al. 2017). Huante et al. (2012) who performed experiments with six tree species reported that AMF inoculation significantly increased the tree growth, especially of slow growing tree species. Also Salto et al. (2020) observed improved growth and increased drought tolerance of *Prosopis alba* inoculated with native AMF under nursery conditions. Similarly, Kapulnik et al. (2010) and Habte et al. (2001) reported about enhanced growth and field performance of *Olea europea* L. and *Acacia koa* after AMF nursery inoculation. Accelerated growth and improved plant health and nutrition of mycorrhized plants can thus help to decrease the growth period under nursery conditions. In addition, inoculation with native versus exotic AMF species should be favored as recently reviewed by Berutti et al. (2016) who summarized the advantages of using native AMF inoculants over the application of exotic ones. Similarly, Requena et al. (2001) and Caravaca et al. (2003) reported about better field establishment of native shrubs after inoculation with native AMF species in studies to restore degraded Mediterranean ecosystems. In this context, *S. africanum*, which best promoted plant growth in both experiments, represents a promising candidate to be used for inoculation approaches. Also consortium 4, resulting in best growth performance of *P. cineraria*, can be recommended especially since the application of AMF consortia over single-strain inocula should be favoured according to the meta-analysis of Hoeksema et al. (2010), which indicate a higher plant response after inoculation with multiple AMF species.

However, there are some prerequisites to integrate AMF as biofertilizers into largescale agricultural and revegetation programs (Koltai 2010, Igiehon and Babalola 2017). The first is the availability of mass production procedures for selected strains of AMF (Vosátka et al. 2013, Berutti et al. 2016). The second is the availability of suitable, preferably local carriers (Barea et al. 1993; Kapulnik et al. 1994; Douds et al. 2006). Moreover, strict quality control is essential to ascertain the absence of soil-borne pathogens. To fulfill these prerequisites, cooperation with industrial partners could be strived to enable AMF to become part of the sustainable management of this region. Alternatively, also farmers should be encouraged to autonomously produce their AMF inocula starting either from a native culture collection if available or from local soils (Symanczik et al. 2018).

## Conclusion

In this study, native AMF from agricultural and natural habitats in the Southern Arabian Peninsula were isolated, propagated, and identified. Some of the AMF strains that were functionally evaluated represent good candidates for biofertilizers, due to their ability to enhance the growth of two local plants, *P. dactylifera* and *P. cineraria*. Considering the statistical results, the following strains were the most promising ones and resulted in better growth performance than NM-control treatments: *C. drummondii* (strain F41), *Di. aurantia* (strain G8), *Di. spurca* (strain K46), *P. simplex* (strain C49), *R. arabicus* (strain F80), *S. africanum* (strain G14), *S. nakheelum* (strain G90), consortium 3, and consortium 4. It is worth mentioning that the superior growth of plants inoculated with *S. africanum* (strain G14) was consistent in both experiments. This study is an important step towards the integration of native AMF into sustainable agriculture and revegetation programs in the Arabian Peninsula and may serve as a model approach for other arid lands.

## Data Availability

'Not applicable' for that section.
